# A Novel and Reliable Rat Model of Autism

**DOI:** 10.3389/fpsyt.2021.549810

**Published:** 2021-03-12

**Authors:** Zhaoyao Qi, Mengke Lyu, Liping Yang, Haiyan Yuan, Yun Cao, Linlin Zhai, Weili Dang, Juan Liu, Fan Yang, Ying Li

**Affiliations:** ^1^Basic Medical College, Henan University of Chinese Medicine, Zhengzhou, China; ^2^Second Clinical Medical College, Henan University of Chinese Medicine, Zhengzhou, China; ^3^The First Affiliated Hospital of Henan University of Chinese Medicine, Zhengzhou, China; ^4^First Clinical Medical College, Henan University of Chinese Medicine, Zhengzhou, China

**Keywords:** fecal microbiota transplantation, autism, rat model, gut microbiota, rat

## Abstract

**Background:** Autism spectrum disorders (ASD) is a complex neurodevelopmental disorder that lacks an ideal animal model to recapitulate the disease state of ASD. Previous studies have reported that transplanting gut microbiota of ASD patients into pregnant mice is sufficient to promote the changes of autism-like behavior in offspring. This study aims to explore whether fecal microbiota transplantation (FMT) can be used as a new method to establish the ASD animal model.

**Methods:** We transplanted the fecal sample extract of ASD children into pregnant rats (rFMT) repeatedly to establish an ASD rat model (oFMT) and compare it with the classical valproic acid (VPA) model (oVPA).

**Results:** First, we reveal that oFMT shows hypoevolutism and typical behavioral characteristics of ASD, consistent with the previous study. Second, the gut microbiota of oFMT mainly consists of *Firmicutes* and *Bacteroidetes*, recapitulating the abnormal gut microbiota of ASD. In oFMT, the abundance of *Lactobacillus* and *Collinsella* increased (*Lactobacillus: oFMT 60.16%, oVPA 64.13%, oCON 40.11%; Collinsella: oFMT 3.73%, oVPA 1.39%, oCON 1.28%*), compared with oVPA, gut microbiota also showed high consistency. Third, the expression of 5-hydroxytryptamine (5-HT) in oFMT serum increased, γ-aminobutyric acid (GABA) and norepinephrine (NE) in oFMT serum decreased. Fourth, the gut microbiota of oFMT also has some ASD characteristic gut microbiota not found in oVPA. Fifth, pregnant rat with VPA showed significant immune activation, while those with FMT showed relatively minor immune activation.

**Limitations:** Although the mechanism of establishing FMT autism rat model (oFMT) has not clearly defined, the data show that the model has high structural validity, and FMT model is likely to be a new and reliable potential animal model of ASD, and will have potential value in studying gut microbiota of ASD.

**Conclusions:** The FMT autism rat model has high structural validity, and the FMT model is likely to be a new and reliable potential animal model of ASD.

## Introduction

Due to technology or ethics limitation, many ASD related studies cannot directly apply to humans. Animal models can be very convenient to study behaviors and mechanisms. However, the lack of animal models that mimic disease states entirely hinders the research and treatment of ASD. Firstly, a useful model validated by exhaustive animal studies by the exposure of valproic acid (VPA) in rodents' uterus shows striking similarities with the behavior, anatomy, and cellular and molecular changes observed in autistic patients. Numerous anatomical studies have also proved that the VPA model can well-recapitulate the central nervous dysplasia of ASD, providing an excellent and valuable tool to study the underlying mechanism of ASD. However, the VPA biochemical induction model has some limitations (1). For example, most ASD patients have not been exposed to the drug at present, and many previous clinical studies reported that VPA exposure in early pregnancy could also cause nerve cell defects and congenital malformations rather than ASD. Secondly, Sharon and his partners (2) not only discovered that gut microbiota from individuals with ASD is sufficient to promote altered behaviors in rat but also provides adequate evidence for the application of gut microbiota intervention in ASD animal models. However, the combination of gut microbiota and ASD animal model needs to be further explored.

Early abnormal construction of gut microbiota may be the critical factor in the ASD pathogenesis, and numerous reports have promoted the study of potential ASD models of gut microbiota. Previous studies have reported that oral administration of the human commensal *Bacteroides fragilis* can improve communication, stereotype, and sensorimotor behavioral deficits in ASD rats (3, 4). Clinical studies showed that gastrointestinal symptoms often occur in ASD children, and their severity is related to the degree of behavioral disorders (5, 6). The pathogenesis of ASD is not clear, but it has proved to be related to the gut microbiota (7, 8). Other studies found differences in intestinal microbial communities between ASD children and control groups (5, 9–11).

Moreover, fecal microbiota transplantation (FMT) is mainly applied for the treatment of gastrointestinal symptoms, such as Clostridium difficile infection (CDI) (12–14) and Crohn's disease (CD) (15). Later, even in patients with severe Crohn's disease and epilepsy, promising results were obtained (16). In recent years, FMT and neurological disorders have become more closely linked. Researchers such as Kang first conducted a clinical trial in 2017 to test this method's effect on ASD treatment which a small open-label clinical trial evaluated the impact of Microbiota Transfer Therapy on gut microbiota composition and ASD symptoms of 18 ASD-diagnosed children. Their results show that FMT is an effective method to change intestinal microorganisms and improve gastrointestinal and behavioral symptoms of ASD (17). In 2019, they also reported the long-term benefits of FMT in the treatment of ASD (18).

Most importantly, Sharon and his colleagues (2) recently reported that transplanting gut microbiota of ASD patients into mice by oral gavage is sufficient to promote autism-like behavior changes in offspring; however, whether FMT can be used as a new method to establish an ASD rodent model remains to be explored. We transplanted the fecal microbiota from children with ASD into pregnant rats (rFMT) by enema inductively established offspring rat model of ASD (oFMT). Based on the similarity of behavior (exercise activities, social behavior, and stereotyped behavior), the serum factors, and the gut microbiota and oVPA which were previously study, we aim to establish ASD rat model by this method.

## Materials and Methods

### Animal

Wistar rats (*n* = 48; female: Male = 2: 1; male: 270–320 g, female: 180–220 g), were purchased at ages 7 weeks from Beijing Vital River Laboratory Animal Technology Company, Beijing, China. (Permit Number: SCXK (Jing) 2016-0006; Animal Certificate Number: 11400700319215), and housed in the laboratory animal center of the Henan University of Chinese Medicine (CM) (Zhengzhou, China). All rats were maintained in an environment with a consistent temperature of 22 ± 2°C, 55 ± 15% humidity, and 12 h light-dark cycle. This study was conducted at Henan University of CM under the guidelines of the National Institutes of Health Guide for the Care and Use of Laboratory Animals approved by the Animal Ethics Committee of Henan University of CM (Permit Number: DWLL 2018030017). The experimental protocol was approved by the ethics committee of The First Affiliated Hospital of Henan University of Chinese Medicine (Permit Number: 2019HL-084-02).

### The Source of Fecal Microbiota Extract

ASD male children (*n* = 8) in the outpatient department of pediatrics and hospitalized in the First Affiliated Hospital of the Henan University of CM were selected for the current study. The subjects involved in the study excluded other extensive developmental disorders or genetically related autism disorders, no special diet, and no drug treatment affecting gastrointestinal function. We adopted protocol from the previous studies (13, 19–21) for the preparation of fecal bacteria liquid. In a sterile environment, the fresh fecal microbiota extract was directly transplanted or prepared into a mixed liquid containing 10% sterile glycerin, and stored at −80°C. Before use, the frozen fecal microbiota extract was thawed in 37°C thermostat water bath and used within 2 h without repeating freezing and thawing. Institutional Review Board (IRB) at The First Affiliated Hospital of Henan University of CM (IRB Number, 2019 HL-084-02).

### Methods

#### Model Establishment

The rats (female: male = 2:1) paired at night. At 07:00 the next day, if the Yin suppository was detected, the female rat was pregnant for 0.5 d. Maternal pregnant rats were randomly divided into the normal group (*n* = 9), FMT group (*n* = 10) and VPA group (*n* = 8) (Maternal Normal, FMT and VPA annotated rCON, rFMT and rVPA, respectively), offspring continued the maternal group (offspring Normal, FMT and VPA, annotated oCON, oFMT and oVPA). rFMT started from 0.5 d during pregnancy to the full moon of the pups. In other words, fecal microbiota was transplanted into rats from 0.5 d after conception and continued until 21 d after delivery. The fecal microbiota extract (5 g/mL) was transplanted at 0.2 ml/100 g every 2 days. In a sterile environment, after iodophor disinfection, the surroundings of the anus. The rat was calm down, fixed, and inverted. Then, the bore diameter 2 mm sterile soft catheter lubricated with sterile glycerin was slowly inserted for 10–12 cm. We slowly injected the fecal microbiota extract, gently pulled out the catheter, and use the sterile gauze to press the anus 3–5 s to prevent flowing backwards. Calm down the animal after the operation. rVPA were intraperitoneally injected with sodium valproate at 600 mg/kg at pregnancy 12.5 d, offspring rats (oVPA) were classical rat model of ASD, normal maternal rats (rCON) and rFMT were given sterile saline with the same method as mentioned above, normal offspring rats (rCON) served as a control group.

#### Model Evaluation

The rats used for behavioral testing were male offspring (28-days-old) randomly selected from oCON, oFMT, and oVPA, respectively. The technicians who performed the test kept quiet and avoided walking. When replacing animals, wiped the inner and bottom surfaces of the test boxes with alcohol. **(i) Social test:** The 3-chambered social test box (60 cm × 45 cm × 25 cm) was used for improved social experiments. A circular social cage (diameter = 12 cm) is placed in the room's lower left (social room). Furthermore, the area (diameter = 16 cm) around the social cage set as a social area. Rats have placed an empty cage in the right room's upper right corner (test 5 min). The rats were in the middle room. We connected the camera to the top of the box to recorded the movement of the rats. The time spent by the rats in the social room was the latent social time, and the time of the rat's head in the social area was the social time. **(ii) Open field, and stereotyped behavior test:** The bottom of the open field box (100 cm × 100 cm × 45 cm) is divided into 25 grids (20 cm × 20 cm). In a quiet environment, the rats were gently placed in the middle grid of the test box. After acclimation for 1 min, the number of rats passing through the grid (one point for both hind limbs passing through the grid) and the number of standing up (one point for each time for both forelimbs standing off the ground) were recorded within 5 min. The level score and vertical score represent the active scores of rats. The stereotyped behavior observation box (50 cm × 50 cm × 45 cm), after 5 min of accommodation, the test time was 15 min, the tester recorded the number and total time of the rats combing by monitoring video. After the behavioral test, the rats were randomly selected and anesthetized with 10 % chloral hydrate (0.3 ml/100 g). Then, blood was collected from the abdominal aorta, and the serum was centrifuged (3,000 r/min, 15 min) after resting for 15 min. Growth and development indicators for the offspring were measured at post-partum (2–21 d).

#### High-Throughput Amplicon Sequencing of the 16S rRNA Gene

Fecal and cecal microbiota samples were collected on ice after anesthesia. Forward primer, 5′-ACTCCTACGGGAGGCAGCA-3′; Reverse primer, 5′-GGACTACHVGGGTWTCTAAT-3′. Gene sequencing analysis was performed using BMK Cloud (www.biocloud.net).

### Statistical Analysis

All data were presented as $\bar{x}$±*s*. Data processing was performed using IBM SPSS Statistics 22.0, *P-value*<*0.05* was considered statistically significant. We first perform the normality test and the homogeneity of variance test on the three sets of quantitative data. If both are in line, then we use one-way analysis of variance (ANOVA test), and Bonferroni test (corrected ***P*** = 0.05/number of comparisons). If the conditions of the one-way analysis of variance did not meet, the rank-sum test (Kruskal-Wallis test) is used, which also corrects the ***P*** = 0.05/number of comparisons.

## Results

### Growth and Development of Offspring

**Figure 1:**
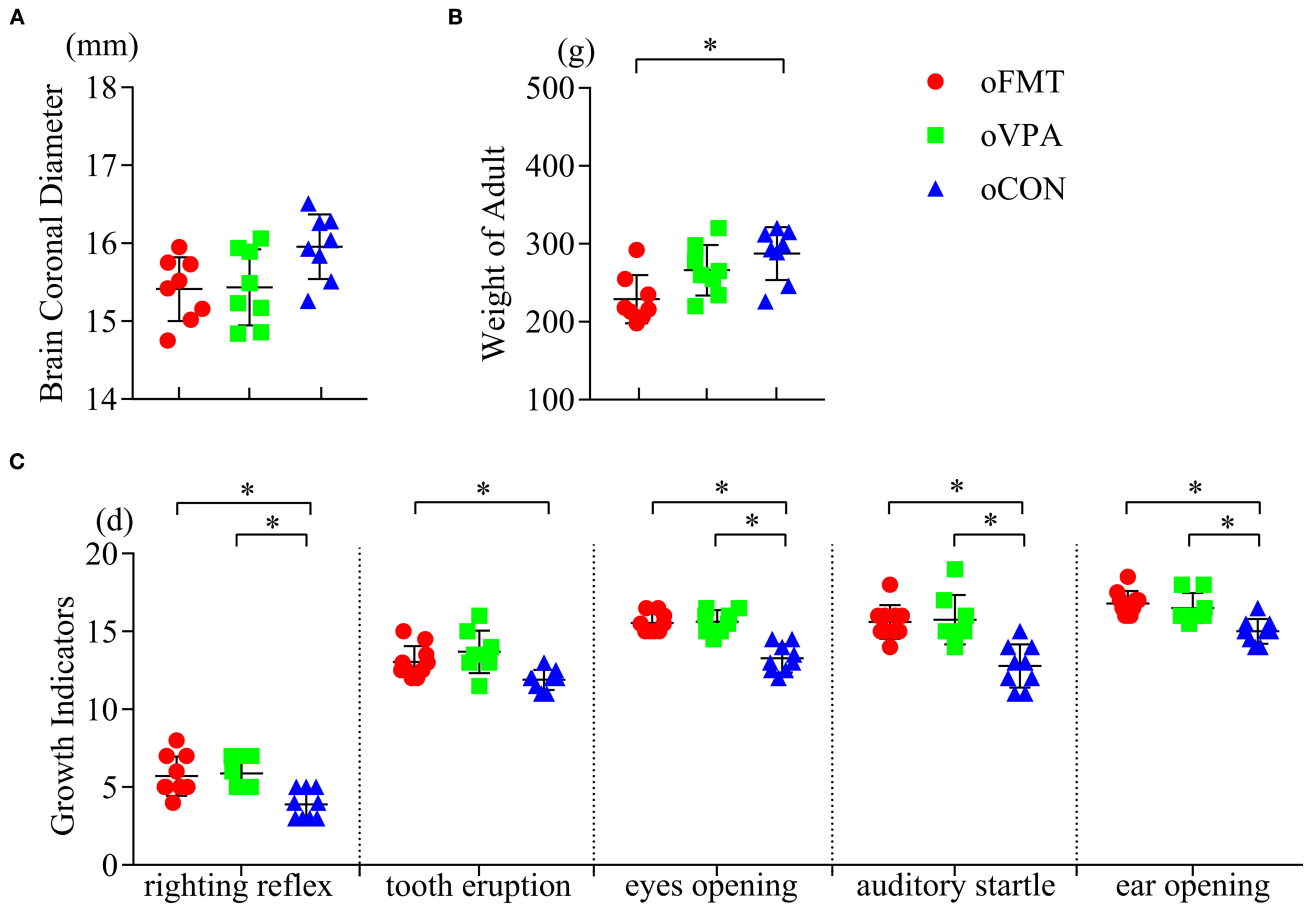
Impact of fecal microbiota transplantation (FMT) on growth and development phenotypes and indicators in offspring rats. The fecal microbiota transplantation group (oFMT) “*P < 0.05” compared with the control group (oCON). (red dot: oFMT; green squares: oVPA; blue triangle: oCON). **(A)** Brain coronal diameter of rats (*n* = 8, each group). **(B)** Bodyweight of adult rats (*n* = 8, each group). **(C)** Growth and development indicators for eye-opening, ear-opening, tooth eruption, auditory startle, and righting reflex (oFMT, *n* = 10; oVPA, *n* = 8; oCON, *n* = 9) (The data were collected from all rats in each litter, then obtain the average value of each litter data. Finally, bring the average value into statistical analysis).

Compare with the control group (oCON), adult weight loss was observed in the oFMT group (Figure 1B) (*P*<0.05). The oFMT showed hypoevolutism, and the delay reached the standard of growth and development indicators, such as eyes opening, ear-opening, tooth eruption, auditory startle, and righting reflex (Figure 1C) (*P*<0.05). Compare with the classical model group (oVPA), the above oFMT indicators are almost consistent with oVPA. No statistical differences were found in coronal brain diameter index among the three groups (Figure 1A).

### FMT Has Induced the Offspring With Characteristic Behavior of ASD

**Figure 2:**
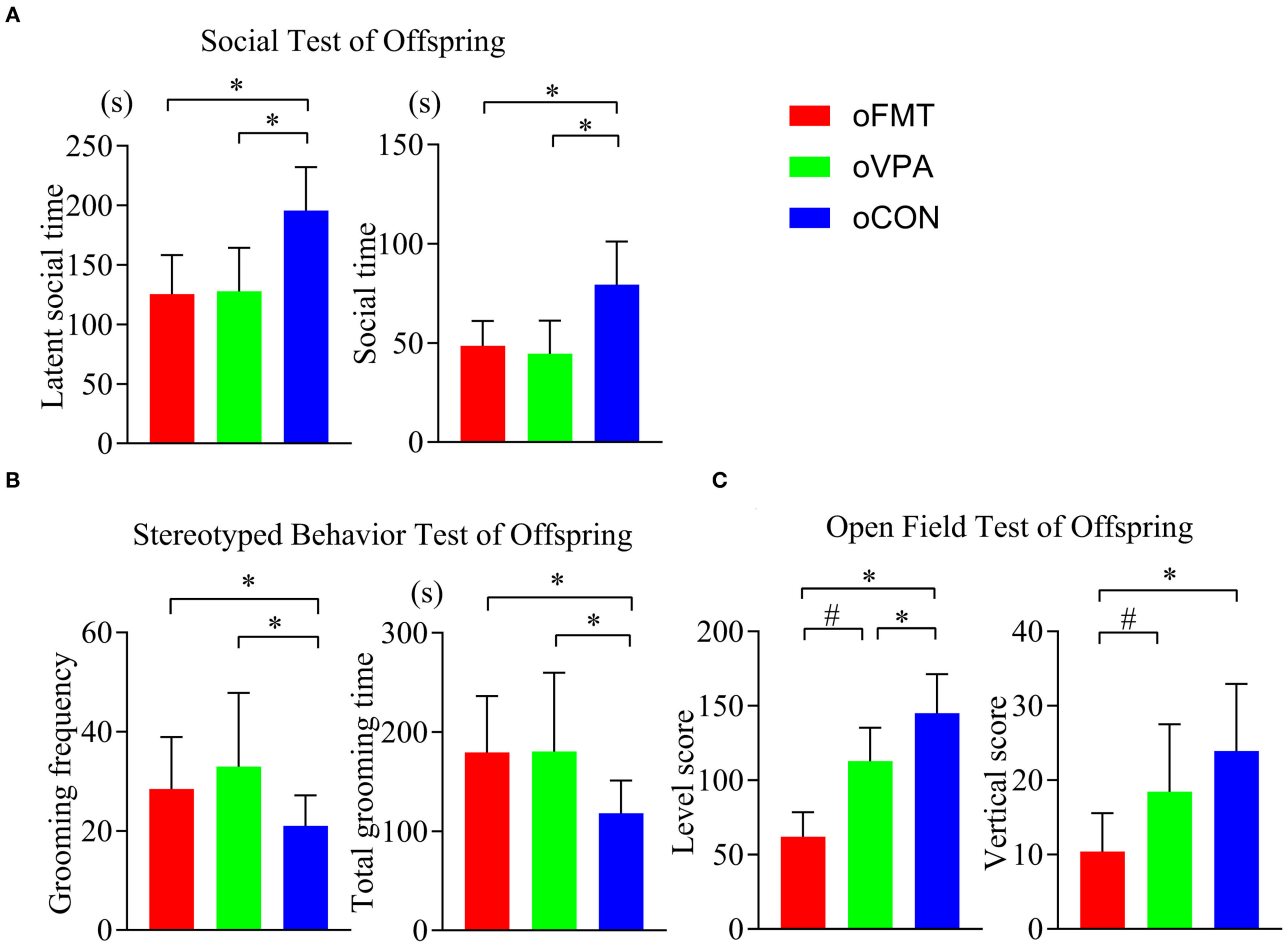
Impact of FMT on behavioral phenotypes and indicators in offspring rats. (red: oFMT; green: oVPA; blue: oCON). “*P < 0.05” compare with the oCON, “^#^***P*** < 0.05” indicates compare with the oVPA. **(A)** Social behaviors test (*n* = 30, each group). **(B)**. Stereotypical behaviors test (*n* = 32, each group). **(C)**. Open filed test (*n* = 20, each group). More than two animals were used in each litter and mixed with effective models for analysis. In other words, non-test experimenters selected rats from each litter by random number table method, and mixed the rats of each group. Finally, we conducted a behavioral test and statistical analysis.

The latent social time and social time of oFMT decreased significantly than the oCON (Figure 2A) (*P*<0.05). Compare with oCON, the stereotypical behaviors of oFMT increased (Figure 2B) (*P*<0.05). The activity score (level score and vertical score) of oFMT decreased significantly (Figure 2C) (*P*<0.05). Compare with oVPA; the above oFMT indicators are consistent with the trend of oVPA. Besides, oFMT showed lack of acting ability than oVPA (Figure 2C) (*P*<0.05).

### Inflammatory Cytokines in Pregnant Rats

**Figure 3:**
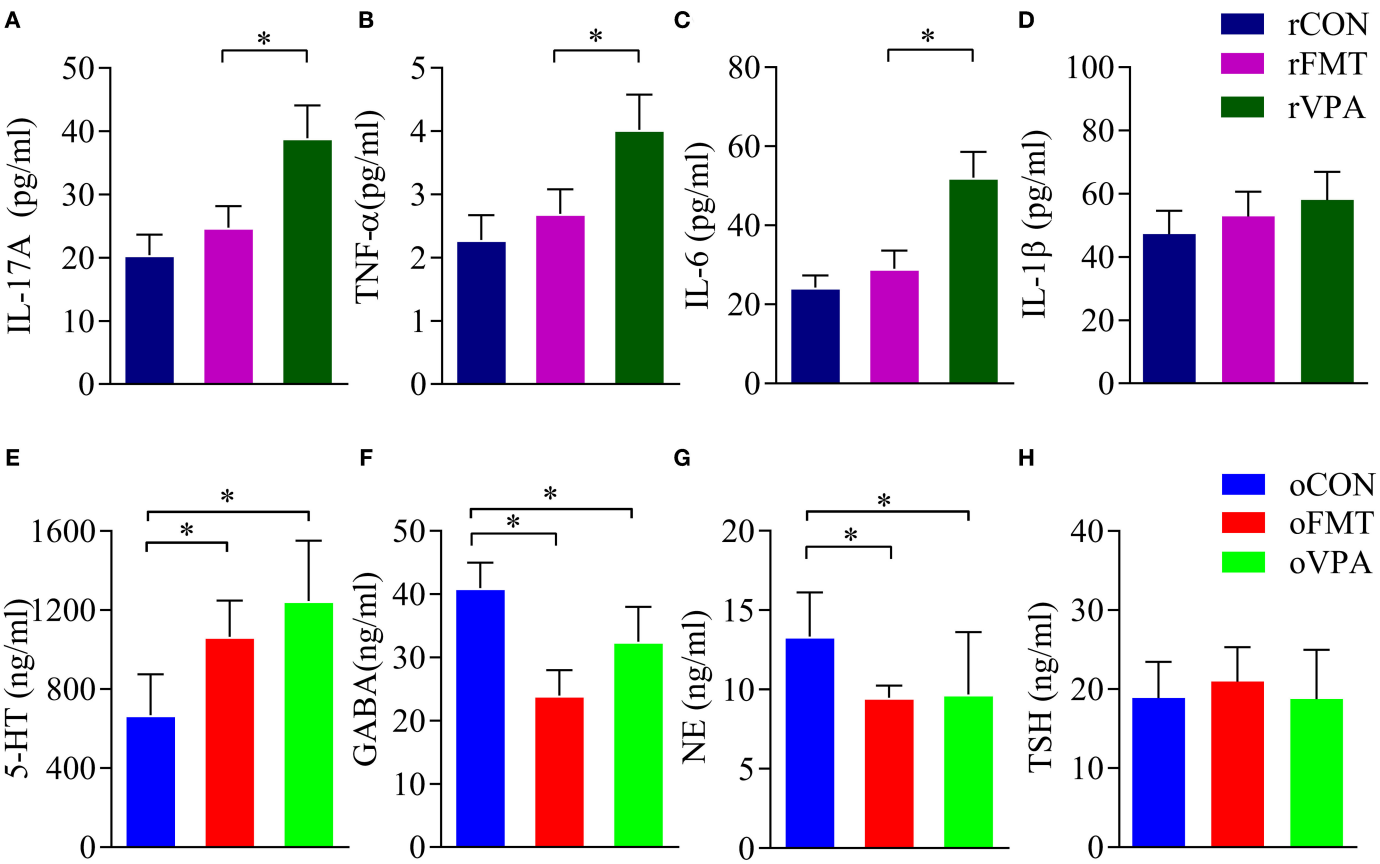
Impact of FMT on serum factor in pregnant and offspring rats. **(A–D)** Serum factor index of pregnant rats. **(E–H)** Serum factor index of offspring rats. Data presented as mean±SD. Data processing was performed using IBM SPSS Statistics 22.0, and statistical significance was established at *P < 0.05 (red: oFMT; green: oVPA; blue: oCON).

Cytokine levels in the maternal serum were detected by ELISA. The levels of IL-17A, TNF-α and IL-6 in the serum of pregnant rats of rVPA group were significantly higher than the rFMT group and the rCON group (Figures 3A–C) (*P* < *0.05*). There was no significant difference between the rFMT group and the rCON group; the levels of IL-1β in each group did not show a significant difference (Figure 3D).

### Neurotransmitters and Hormones in the Offspring

Compared with oCON, in oFMT, the content of 5-HT in serum increased significantly (Figure 3E) (*P*<0.05); GABA content in oFMT and oVPA was significantly lower than the oCON, and GABA and NE content in oFMT was the lowest (Figures 3F,G) (*P* < *0.05*). The thyroid-stimulating hormone (TSH) was also increased (Figure 3H), but this is not supported by statistics. Compared with oVPA, the above oFMT index is consistent with oVPA.

### The Changes of Gut Microbiota Among oCON, oFMT, and oVPA Based on the 16S rRNA Data

**Figure 4:**
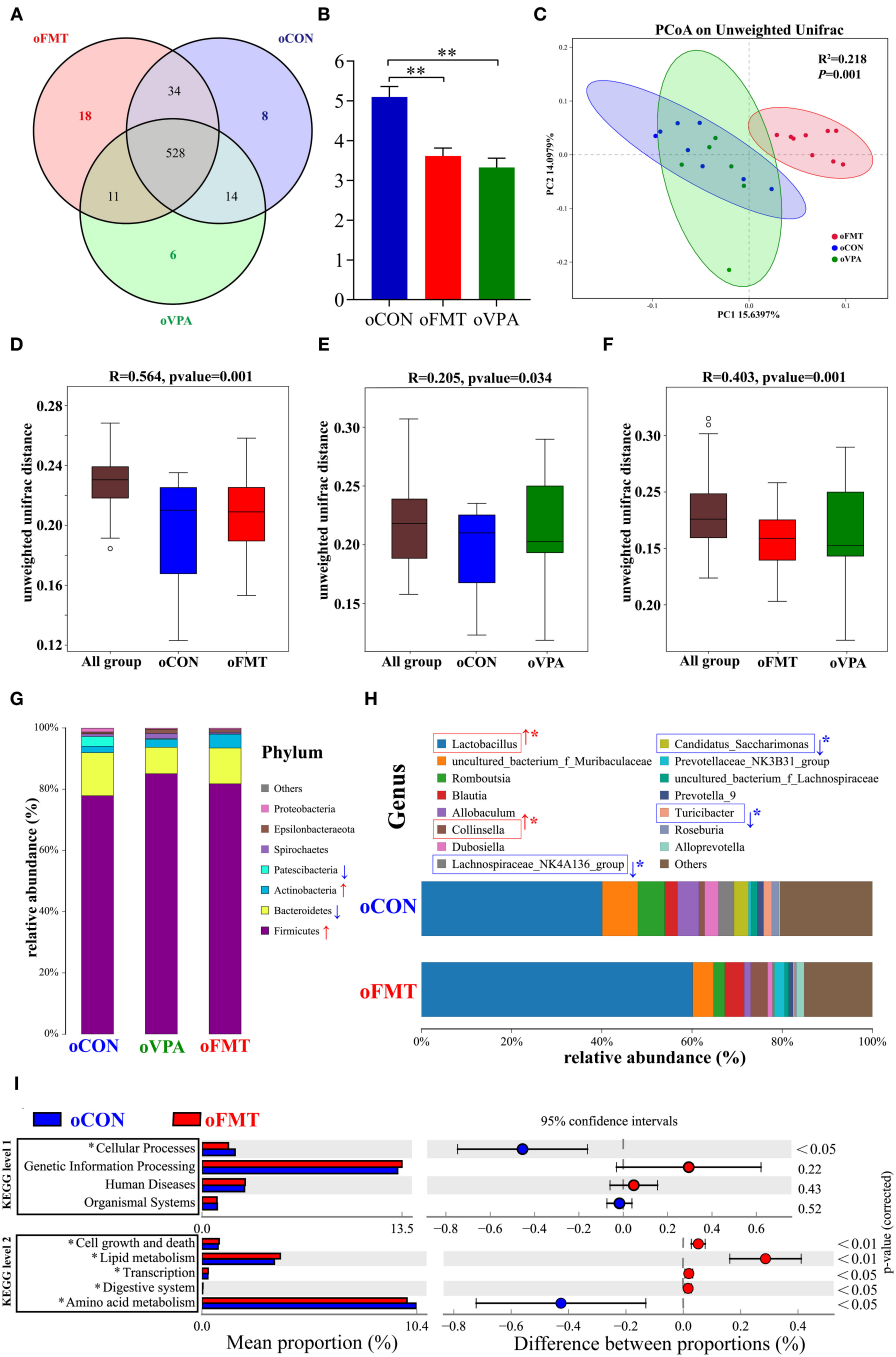
The gut microbiota among oCON, oFMT, and oVPA according to the 16S rRNA data. Impact of FMT on gut microbiota (cecal and fecal microbiota) in offspring rats (oFMT, *n* = 9; oVPA, *n* = 6; oCON, *n* = 8) (red: oFMT; green: oVPA; blue: oCON). **(A)** Venn diagram of the observed OTUs among oCON, oFMT, and oVPA. **(B)** The Shannon index (one-way analysis of variance test) of the gut microbiome among oCON, oFMT, and oVPA according to all OTUs data. **P < 0.01 compare with the oCON. **(C)** Principal Coordinates Analysis (PCoA) (Unweighted Unifrac algorithm) based on all OTUs. The inter-group difference test analyzed by PERMANOVA analysis **(D–F)**. Analysis of similarities (ANOSIM) was used to test whether there was a significant difference in Beta diversity between samples in two different groups. The vertical axis represents Beta distance. The box chart above “All group” represents the Beta distance data of samples between all groups, while the box chart on the right represents the Beta distance data of samples within different groups. The closer R-value to 1.0, the greater the inter-group difference than the intra-group difference. The reliability of the test is significant when *P-*value < 0.05. **(G)** The gut microbiota species distribution analysis between oCON and oFMT in the Phylum. Red “↑” shows the relative abundance increased, blue “↓” means decreased compare with oCON. **(H)** The gut microbiota species distribution analysis in the Genus. “^*^” indicates that the microbiota has a significant difference in microbial community abundance between oCON and oFMT (Metastats analysis) (*P < 0.05). Red “↑” shows the relative abundance increased, and blue “↓” means decreased. **(I)** Difference analysis of KEGG metabolic pathway in oCON and oFMT at the first and second levels. The left is the proportion of the abundance of different functions in two groups of samples; in the middle is the proportion of the difference in the abundance of different functions in the 95% confidence interval; on the far right is the corrected *P*-value.

The changes in gut microbiota composition among oCON, oFMT, and oVPA were explored. As shown in Figure 4A, the Venn diagram shows the 18 unique operational taxonomic units (OTUs) of oFMT, the 6 unique OUTs of oVPA, and the 8 unique OTUs of oCON. There were 528 OTUs in the three groups (Figure 4A). The species diversity (Shannon index) in the oFMT and oVPA were significantly lower than that in the oCON (Figure 4B) (***P*** < 0.01). Principal coordinate analysis (PCoA) of the microbiota based on the unweighted UniFrac distance metrics for oCON, oFMT, and oVPA (Figure 4C). Which was performed to study the degree of similarity of microbial communities in the three groups. The results showed that the microbiota composition of oVPA was more uneven than that of oFMT. The oFMT was significantly different from the oVPA and oCON (***R***^2^ = 0.218, ***P*** < 0.05).

Analysis of similarities (ANOSIM) was used to test whether there was a significant difference in Beta diversity between samples in the two groups. The vertical seat represents Beta distance. The box chart above “All group” represents the Beta distance data of samples between all groups, while the box chart on the right represents the Beta distance data of samples within different groups. The closer the R-value obtained by ANOSIM analysis to 1, the greater the difference between groups than the difference within groups; the smaller the R-value, there will be no significant difference between and within groups. The reliability of the test is significant when ***P*** < 0.05 (Figures 4D–F). The results show that the microbiota composition of oFMT was more different than that of oVPA, comparing with oCON. The oFMT was different from the oVPA and oCON (*R*^2^ = 0.218, ***P*** < 0.05). This is consistent with the result of the PCoA analysis as a whole.

The gut microbiota species distribution analysis among oCON, oFMT, and oVPA in the Phylum. *Firmicutes* and *Bacteroidetes* are dominant in oFMT and oVPA (*Firmicutes* and *Bacteroidetes*: oFMT 93.45%, oVPA 93.68%, and oCON 91.93%). Compared with oCON, the relative abundance of *Firmicutes* and *Actinobacteria* increased (*Firmicutes*: oCON 77.87%; oFMT 81.75%, oVPA 85.06%), and the relative abundance of *Bacteroidetes* and *Patescibacteria* decreased in oFMT and oVPA (*Bacteroidetes*: oCON 14.06%; oFMT 11.69%, oVPA 8.62%) (Figure 4G).

**Figure 5:**
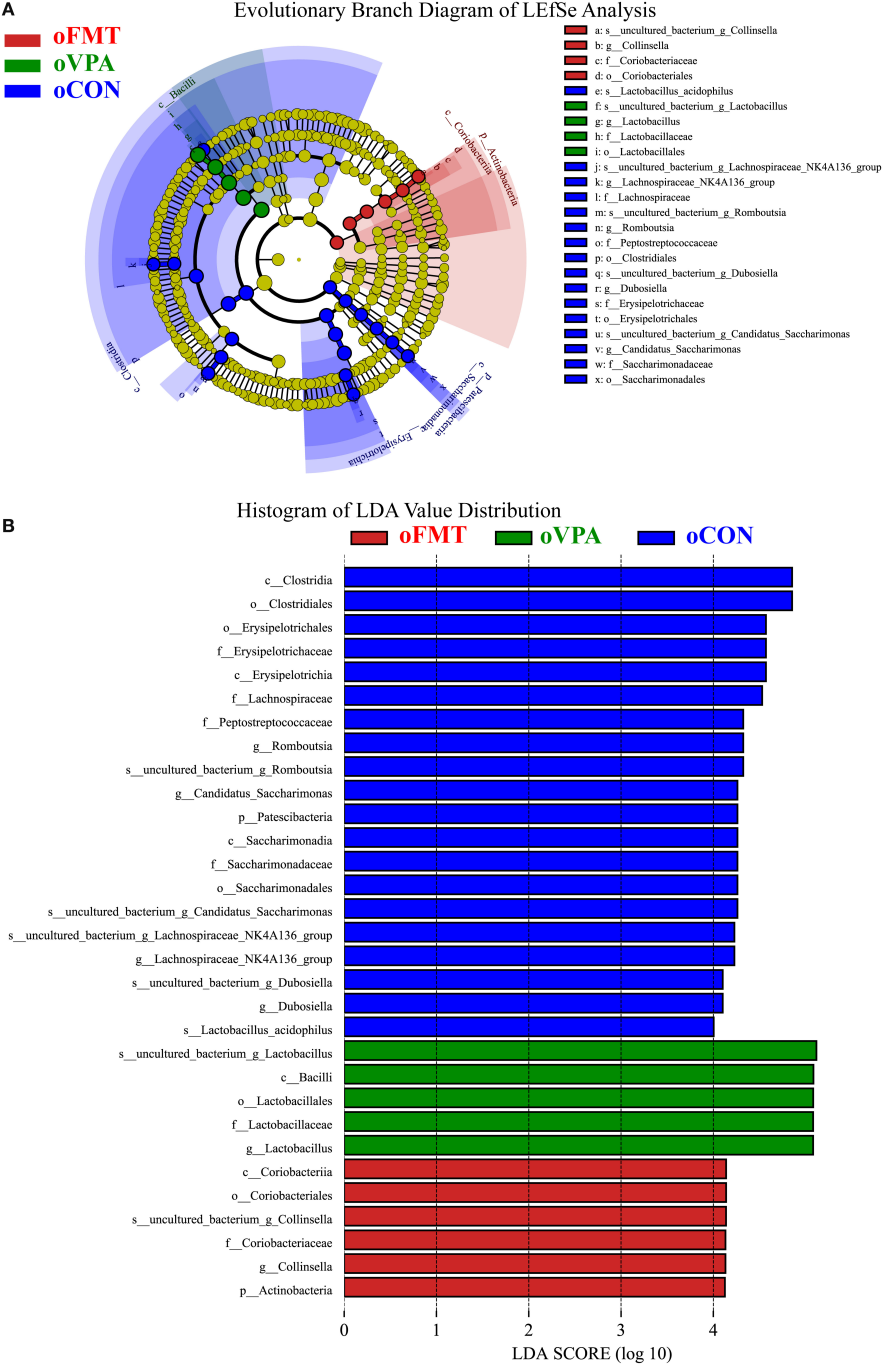
**(A)** Evolutionary Branch Diagram of LEfSe Analysis. **(B)** Histogram of LDA Value Distribution. Linear Discriminant Analysis (LDA) Effect Size (LEfSe) analysis of gut microbiota in offspring rats among oCON, oFMT, and oVPA according to the 16S rRNA data. The yellow nodes represent the microbiota that did not play an essential role in different groups, and no biomarker meeting the requirements. The histogram of LDA value distribution and the evolutionary branch diagram of LEfSe analysis show the species whose LDA Score is higher than the set value (LDA score >4.0, ***P*** < 0.05). The histogram's length represents the impact size of different species (LDA Score), and different colors represent the species in different groups (red: oFMT; green: oVPA; blue: oCON).

Figure 5 shows the Linear Discriminant Analysis (LDA) Effect Size (LEfSe) analysis of gut microbiota in offspring rats among oCON, oFMT, and oVPA, according to the 16S rRNA data. The histogram of LDA value distribution and the evolutionary branch diagram of LEfSe analysis show the species whose LDA Score is higher than the set value (LDA score >4.0, ***P*** < 0.05). The histogram's length represents the impact size of different species (LDA Score), and different colors represent the species in different groups. LDA and LEfSe analysis show that *Actinobacteria* was significantly enriched in oFMT, and *Firmicutes* was significantly enriched in oVPA at the Phylum level. However, the results show that *Patescibacteria* and *Firmicutes* were significantly enriched in oCON at the Phylum level. Besides, we also found that the abundance of *g_Collinsella* in oFMT and *g_Lactobacillus* in oVPA were higher. The abundance of *g_Dubosiella, g_Lachnospiraceae_NK4A136_group*, and *g_Romboutsia* were enriched in oCON. To sum up, this finding indicates that the specificity of gut microbiota in oFMT was higher than that in oVPA.

### The Changes in Gut Microbiota Between oCON and oFMT According to the 16S rRNA Data

The gut microbiota species distribution analysis between oCON and oFMT in the Genus, *Lactobacillus* is dominant (oCON 40.11%, and oFMT 60.16%). Compared with oCON, the relative abundance of *Lactobacillus* and *Collinsella* increased (*Collinsella*: oCON 1.28%, oFMT 3.73%), and the relative abundance of *Lachnospiraceae_NK4A136_group, Candidatus_Saccharimonas* and *Turicibacter* decreased (*Lachnospiraceae_NK4A136_group*: oCON 3.47%, oFMT 0.37%; *Candidatus_Saccharimonas*: oCON 3.19%, oFMT 0.07%; *Turicibacter*: oCON 1.84%, oFMT 0.33%;) (Figure 4H). To investigate the differences in microbial community abundance between oCON and oFMT, *t*-test was performed on species abundance data at the Genus level by Metastats analysis. Finally, the species causing the difference in sample composition between the two groups were screened according to the corrected *P*-value (***P*** < 0.05). In conclusion, the relative abundance of *Lactobacillus* in oFMT was significantly increased compared with oCON, suggesting that there may be an imbalance in the bacterial community structure.

The 16S RNA sequencing can also be used for functional prediction analysis of gut microbiota. Difference analysis of KEGG metabolic pathway in oCON and oFMT were executed at the first and second levels. The left is the proportion of the abundance of different functions in two groups of samples; in the middle is the proportion of the difference in the abundance of different functions in the 95% confidence interval; on the far right is the corrected *P*-value (***P*** < 0.05). We compared the predictive functions of gut microbiota and revealed the differences in microbial functions between oFMT and oCON based on the KEGG module. Meanwhile, compared with the oCON, some KEGG pathways based on level 1 and level 2 in FMT were different (Figure 4I). For example, Cellular Processes (level 1) and Amino Acid Metabolism (level 2) were lower and Cell growth and death, Lipid metabolism, Transcription, and Digestive system were higher in the oFMT compared with oCON.

### The Difference of Gut Microbiota Between oFMT and oVPA Based on the 16S rRNA Data

**Figure 6:**
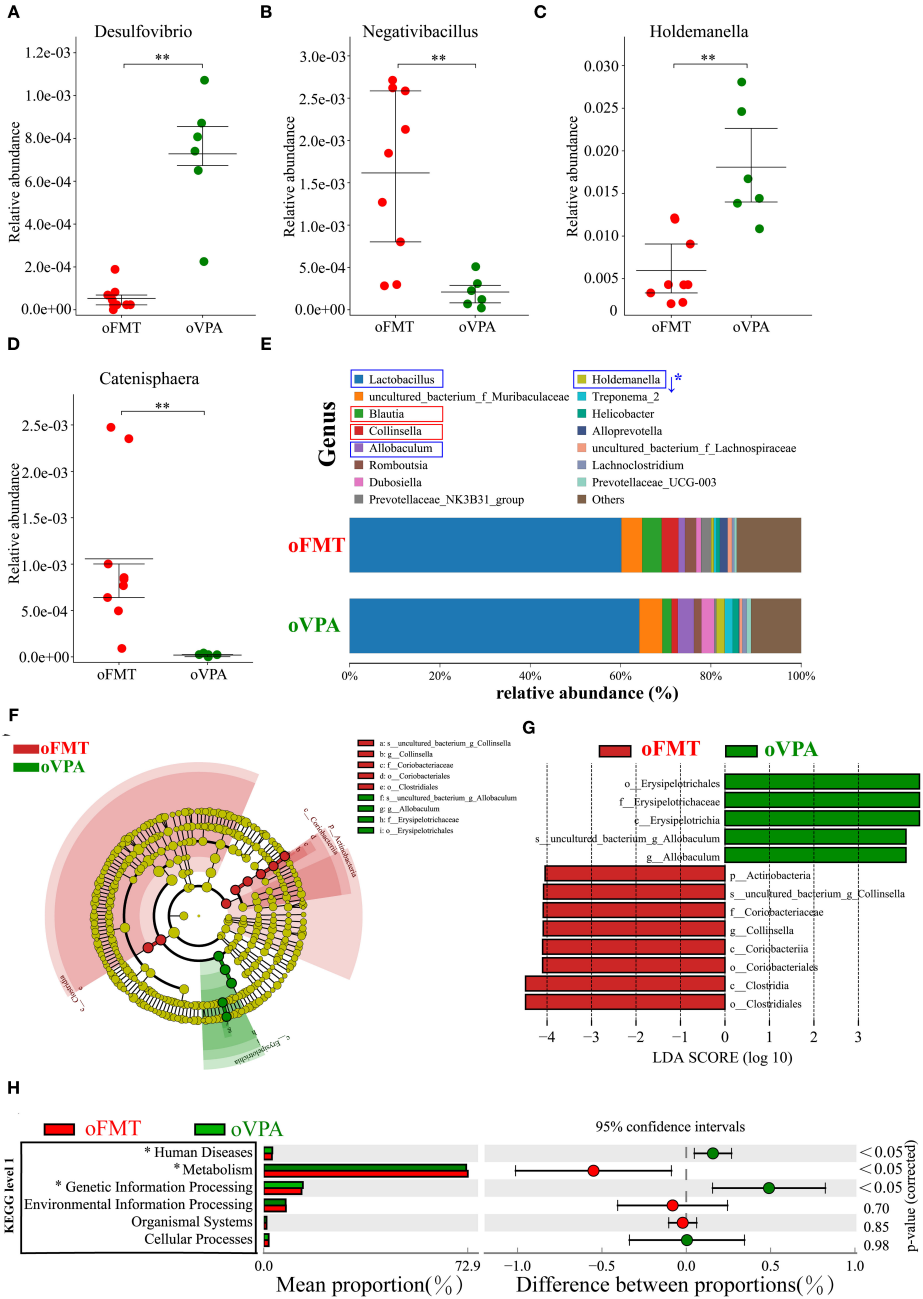
The changes in the gut microbiota composition between oFMT and oVPA according to the 16S rRNA data. Impact of FMT on gut microbiota (cecal and fecal microbiota) in offspring rats (oFMT, *n* = 9; oVPA, *n* = 6) (red: oFMT; green: oVPA). **P < 0.01 compare with the oCON. **(A–D)** To investigate the differences in microbial community abundance between oFMT and oVPA, *t*-test was performed on species abundance data at the Genus level by Metastats analysis. Finally, the species causing the difference in sample composition between the two groups were screened according to the corrected *P*-value. **(E)** The gut microbiota species distribution analysis in the Genus. The microbiota has a significant difference in microbial community abundance between oFMT and oVPA (Metastats analysis) (*P < 0.05). Red “↑” shows the relative abundance increased, and blue “↓” means decreased. **(F,G)** The histogram of LDA value distribution and the evolutionary branch diagram of LEfSe analysis show the species whose LDA Score is higher than the set value (LDA score >4.0, ***P*** < 0.05). The histogram's length represents the impact size of different species (LDA Score), and different colors represent the species in different groups. **(H)** Difference analysis of KEGG metabolic pathway in oFMT and oVPA at the first level. The left is the proportion of the abundance of different functions in two groups of samples; in the middle is the proportion of the difference in the abundance of different functions in the 95% confidence interval; on the far right is the corrected *P*-value.

To investigate the differences in microbial community abundance between oFMT and oVPA, *t*-test was performed on species abundance data at the Genus level (Metastats). Finally, the species causing the difference in sample composition between the two groups were screened according to the corrected *P*-value (***P*** < 0.05). The data indicated that the relative abundances of *Negativibacillus* and *Catenisphaera* were increased, while those of *Desulfovibrio* and *Holdemanella* in oFMT were decreased, compared with those of oVPA (Figures 6A–D). The gut microbiota species distribution analysis between oVPA and oFMT in the Genus, *Lactobacillus* is in a dominant position (oVPA 64.13%, oFMT 60.16%). Compared with the oVPA, the relative abundance of *Blautia, Collinsella, Prevotellaceae_NK3B31_group, Alloprevotella*, and *Romboutsia* increased (*Collinsella*: oVPA 1.39%, oFMT 3.73%), and the relative abundance of *Allobaculum, Dubosiella, Holdemanella, Treponema_2*, and *Helicobacter* (Metastats analysis, corrected ***P*** < 0.05) decreased (Figure 6E). Compare with oVPA, the microbiota species distribution of oFMT was similar to oVPA. However, oFMT also has some characteristic microbiota, which may be the possible reason for the difference between oFMT and oVPA.

The histogram of LDA value distribution and the evolutionary branch diagram of LEfSe analysis show the species whose LDA Score is higher than the set value (LDA score >4.0, ***P*** < 0.05) (Figures 6F,G). The length of the histogram represents the impact size of different species (LDA Score), and different colors represent the species in different groups. LDA and LEfSe analysis show that *g_Collinsella* and *o_Clostridiales* were significantly enriched in oFMT, and *g_Allobaculum* was significantly enriched in oVPA from the Phylum to Genus level. In other words, the abundance of *g_Collinsella* and *o_Clostridiales* in oFMT and *g_Allobaculum* in oVPA were high. To sum up, the specificity and diversity of gut microbiota in oFMT was higher than that in oVPA. This result suggested that the composition of gut microbiota in oFMT had undergone greater changes by ASD gut microbiota FMT.

Difference analysis of KEGG metabolic pathway in oVPA and oFMT at the first level. The left is the proportion of the abundance of different functions in two groups of samples; in the middle is the proportion of the difference in the abundance of different functions in the 95% confidence interval; on the far right is the corrected *P*-value (***P*** < 0.05) (Figure 6H). Compared with the oVPA, some KEGG pathways based on level 1 in oFMT were different. For example, Human Discases and Genetic Information Processing were lower and the metabolism was higher in the oFMT compared with oCON.

## Discussion

The gut microbiota affects many essential host functions, including the immune response and the nervous system. It has been reported that patients with ASD and their families have been diagnosed with immune abnormalities; however, the causal relationship between pregnant mothers' immune status and the risk of having children with ASD is still unclear (22, 23). In epidemiological studies and animal models, prenatal immune attacks (such as bacteria) are associated with behavioral disorders in later generations (24). The gut microbiota was also closely related to maternal immune activation. A previous study showed that gut commensal bacteria with a tendency to induce T helper 17 cells may increase the risk of neurodevelopmental disorders in the offspring of pregnant women with immune system activation due to infection or autoimmune inflammation syndrome (25). After the pregnant rats in this study received the ASD gut microbiota, the inflammatory factors (levels of change were insignificant compared with oCON) suggested that there was no significant maternal activation. However, the oVPA immune factor is up-regulated, indicating that it may have immune activation (compared with oCON).

In this study, the ASD rat model was established and evaluated by FMT. Our data show that the gut microbiota of children with ASD transplanted into sterile pregnant rats is sufficient to promote autistic-like behavior in the offspring. In this case, the maternal intervention can also cause behavioral abnormalities in the offspring, suggesting that there may be a new pathway. 5-HT and γ-aminobutyric acid (GABA) play important roles in developing and progressing neurological function during pregnancy and early childhood. Importantly, gut microbiota may alter GABA and 5-HT levels. The distribution of gut microbiota in FMT and VPA models shows striking agreement. Such as the gut microbiota species distribution analysis between oVPA and oFMT in the Genus, *Lactobacillus* is in a dominant position. However, oFMT also has some characteristic microbiota, which may be the reason for the difference between oFMT and oVPA. For example, at the Genus level, the data indicated that the relative abundances of *Negativibacillus* and *Catenisphaera* were increased, while those of group *Desulfovibrio* and *Holdemanella* in the oFMT were decreased, compared with those of the oVPA.

A promising animal model must show confronting effectiveness (similar to human symptoms), structural effectiveness (similar to underlying mechanisms) and predictive effectiveness (expected response to effective human therapy) (26). Regarding face validity, gut microbiota ASD model rats (oFMT) showed developmental abnormalities, and more importantly, it exhibits ASD core behavioral characteristics. First, previous studies reported that VPA model rats showed delayed maturation (delayed eye-opening), lower body weight, and delayed motor development (27). The physical development of FMT treated rats delayed because they showed lower body weight, delayed the time of eyes opening, ear-opening, tooth eruption, auditory startle, and righting the reflex. This study speculated that it was related to delayed development. Secondly, the diagnosis of ASD based on three behavior patterns frequently observed by ASD. (i) Exercise and exploration activities reduced, children with ASD have lower environmental exploration behaviors, and the acting ability in ASD animals also reduced (28). (ii) Repeated rigid behavioral activities, for example, children with ASD repeatedly played with fingers or the same toy, and ASD animals showed repeated modification of hair (29, 30). (iii) Social behavior activity, its reduction (social disorder), is a core feature of ASD (27, 31, 32). The oFMT showed behavioral defects, such as decreased activity ability (reduced activity score in open-field tests), increased repetitive, stereotyped behaviors (increased frequency and time of repeated grooming), and social barriers (decreased exploration activities for strange rats) in the improved three-box social experiment. These behavioral manifestations suggest that oFMT has autism-like behavior, indicating that fecal bacteria transplantation into the ASD rat model has good faced validity.

In terms of structural validity, increasingly research data show that gut microbiota was closely related to neurological diseases. For example, gut microbiota plays a role in guiding and promoting brain development (33); abnormal early colonization of gut microbiota may be an essential factor inducing ASD symptoms (34). Furthermore, a number of studies have also reported that the gut microbiota in children with ASD has changed significantly. This study has also observed consistent results. For example, oFMT (Phylum) increases to *Firmicutes*, decreases to *Bacteroidetes*, and increases to *Firmicutes/Bacteroidetes* (35–37); at the genus level, the increase in *Lactobacillus* (35, 36), and *Collinsella* also increase (35). These significant changes in gut microbiota are consistent with previous studies (35–37). Compared with the VPA model, the gut microbiota ASD rat model also shows consistency in gut microbiota. For example, *Firmicutes* and *Bacteroidetes* are dominant in gut microbiota analysis, and the changing trend is the same, which is the well-agreement with previous studies (38). The composition of the gut microbiota of oFMT is similar to that of oVPA.

First, based on 16S rRNA data, the changes in gut microbiota composition among oCON, oFMT, and oVPA were explored. The species diversity in the oFMT and oVPA was significantly lower than that in the oCON. The results showed that the diversity of gut microbiota in offspring rats was impaired. This may be the source of behavioral anomalies in both oFMT and oVPA. PCoA analysis was performed to study the degree of similarity of microbial communities across the three groups. To increase the reliability of the difference analysis, ANOSIM analysis was used to test whether there was a significant difference in diversity between samples in different two groups. The data shows that the microbiota composition of oVPA was more uneven than oFMT. The oFMT was different from the oVPA and oCON

Second, the gut microbiota species distribution analysis among oCON, oFMT, and oVPA in the Phylum. *Firmicutes* and *Bacteroidetes* are in a dominant position in oFMT and oVPA. Compared with oCON, the relative abundance of *Firmicutes* and *Actinobacteria* increased, and the relative abundance of *Bacteroidetes* and *Patescibacteria* decreased in oFMT and oVPA. LDA and LEfSe analysis gut microbiota shows that *Actinobacteria* was significantly enriched in oFMT and *Firmicutes* was significantly enriched in oVPA at the Phylum level. Moreover, we also found that the abundance of *g_Collinsella* in oFMT and *g_Lactobacillus* in oVPA were higher. The abundance of *g_Dubosiella, g_Lachnospiraceae_NK4A136_group*, and *g_Romboutsia* were enriched in oCON. We can draw a conclusion that the specificity of gut microbiota in oFMT was higher than that in oVPA. This suggested that the composition of gut microbiota in oFMT had undergone significant changes. To sum up, the gut microbiota composition of oFMT was similar to oVPA, but oFMT also had some unique features; for example, the marker bacteria analysis group was actinomycetes (Phylum level), which was different from oVPA and oCON. Which may be the reason for the specific expression of oFMT gut microbiota structure.

Third, we also found a very interesting phenomenon, oFMT and oVPA are two different ways to establish the ASD rat model, but the bacterial community composition of both at the Genus level is very similar. In particular, there has a coincidentally significant increase in *Lactobacillus* compare with oFMT, which is considered to be probiotics. There have been similar reports (35) about the elevation of *Lactobacillus* in the gut microflora structure of ASD patients, but still, no attention has been paid to it. In conclusion, through comparing the two modeling methods, the increase of *Lactobacillus* may not be accidental but may contain a complex relationship of bacterial flora structure, which needs to be further explored. Surprisingly, the oFMT also has ASD characteristic gut microbiota that oVPA does not have. For example, *Prevotellaceae* (39, 40), *Clostridiaceae* (41), *g_Collinsella* (35), *p_Actinobacteria, f_Coriobacteriaceae, o_Coriobacteriales*, and *o_Clostridiales, c_Clostridia*. These findings indicate that oFMT may have ASD characteristic gut microbiota that oVPA cannot simulate, which is worthy of the future research direction.

The communication mechanism between gut microbiota and the brain is still unclear, and researchers are paying close attention to elucidate the relationship between gut microbiota and the nervous system. The alteration of the gut microbial spectrum is associated with the abnormal metabolic activity of ASD (42). It is well-established that the gut tract contains trillions of bacteria that regulate the production of various signaling molecules in a host, including, 5-HT, GABA, NE and other hormones and neurotransmitters. The neurotransmitter is an endogenous metabolite that acts as a chemical messenger to carry, amplify and regulate signals between neurons and other human cells. According to their functions, they can be divided into excitatory neurotransmitters (such as NE), and inhibitory neurotransmitters (such as GABA and 5-HT). These substances play an important role in the normal life activities of the body and maybe the bridge or messenger between the gut microbiota and neurodevelopment. 5-HT is a critical step in neuronal development, such as cell proliferation, differentiation, migration, apoptosis, synapse formation, and play an important role in neuronal and glial cell development. The increase in serum 5-HT is related to autism (43). At critical developmental stages, one-third of children with autism have elevated 5-HT in their peripheral blood (44). 5-HT also has the function of regulating gut microbiota (45, 46).

Serum 5-HT levels of oFMT were higher than oCON, which might be related to the neurological dysfunction of ASD. Some studies have reported elevated TSH levels in children with ASD (47), while other studies have confirmed the clinical diagnostic significance of TSH (48). Moreover, GABA (gamma-aminobutyric acid) is a quiet neurotransmitter that inhibits neuronal reflexes. Brain studies in excitatory populations have identified imbalances in GABA receptors. Lower NE is closely related to memory disorders, negative mental state, and neurodevelopmental disorders. Inadequate GABA levels, or dysfunction of GABA receptor, play a role in the excitatory factors of autism and attention deficit hyperactivity disorder. Serum GABA and NE levels of oFMT were significantly lower than oCON, which might be related to the abnormal gut microbiota of ASD. Previous studies have found that Bacteroides (such as Bifidobacterium) produce large amounts of GABA. Analysis of healthy human feces samples has demonstrated that the GABA-producing pathway is actively expressed by species such as Bacteroides, Parabacteroides, and Escherichia (49). oFMT (Phylum) increases to Firmicutes, decreases to Bacteroidetes, and increases to Firmicutes/Bacteroidetes (35–37). Both the abnormal structure of gut microbiota in ASD and changes in the metabolism of neurotransmitters are necessary for revealing the causes of ASD. The up-regulation of serum 5-HT level and down-regulation of GABA and NE may lead to behavioral and physiological changes in offspring rats. All these signified that the microbiota could cause abnormal neurotransmitter metabolism, which was probably the critical process of model establishment and needed further data to verify.

Regarding the prediction validity, numerous studies have reported that correcting the abnormality of gut microbiota can alleviate ASD animals (3, 50) or children (17, 18, 36). However, unfortunately, our experiment did not perform subsequent disproportional assays, such as transplantation of healthy human gut microbiota or probiotic therapy. The sampling of gut microbiota in ASD animal experiments includes cecal and fecal microbiota samples (2, 38). Thus, this study selected cecal and fecal microbiota samples. The 16S RNA sequencing can also be used for functional prediction analysis of gut microbiota. Difference analysis of KEGG metabolic pathway in oCON and oFMT at the first and second levels. Compare with the oCON, some KEGG pathways based on level 1 and level 2 in FMT were different. For example, Cellular Processes (level 1) and Amino Acid Metabolism (level 2) were lower, and Cell growth and death, Lipid metabolism, Transcription, and Digestive system were higher in the oFMT comparing with oCON. Functional prediction analysis suggests that the bacterial function of the oFMT may be diminished. Meanwhile, compared with the oVPA, some KEGG pathways based on level 1 in oFMT were different. For example, Human Discases and Genetic Information Processing were lower, and Metabolism was higher in the oFMT compared with oCON. Functional prediction analysis showed that the bacterial function of the oFMT and oVPA might be different.

VPA is an antiepileptic drug (51) and an emotion stabilizer (52), which inhibits the activity of histone deacetylase (HDAC). VPA rodent model of ASD is currently the most effective and reliable ASD animal models and has widely used in ASD research. However, it also has certain limitations. Currently, most ASD patients have not exposed to the drug, and its incidence rate is still rising sharply, reaching 1/59 (53), indicating that VPA exposure is only a critical pathogenic factor of ASD. Besides, many clinical reports also link VPA exposure to early pregnancy with neural cell defects and congenital malformations other than ASD (54, 55), indicating that the induction result of VPA is not single. Therefore, the effectiveness of the VPA rodent model may be limited to autism caused by exposure to drugs with HDAC inhibitory activity (1), which has excellent application valued in the study of ASD neurodevelopmental abnormalities and origin. Meanwhile, many promising models of ASD gene (such as Shank3, MeCP2, and FMR1) may also face similar situations with VPA rodent models. Although the FMT model can not bridge these limitations, it may provide new ideas for establishing the ASD animal model. The development of intestinal microflora is synchronous with the development of children's brains. The onset stage of ASD is similar to the time node of intestinal microflora development (7, 34). FMT model simulates the face validity (development and behavior) of ASD, especially in gut microbiota. FMT model also has strong structural validity (compared with ASD patients and VPA model); meanwhile, it has profound abnormal performance in serum immunity. Compared with the VPA model, the FMT model did not have a solid research foundation, but the FMT model to provide a new choice of ASD research, primarily it can provide a reliable model tool for studying ASD cases of severe gastrointestinal symptoms or gut microbiota disorders.

## Limitations

According to our data, the FMT model is likely to be a new and reliable potential animal model of ASD and has potential value in studying the gut microbiota of ASD. However, our experiments also have some limitations. First, this study only emphasizes the effectiveness of the model. Which needs a more abundant and rigorous experimental design to supplement the model formation rate in the future. Second, our modeling operation takes a long time to transplant fecal bacteria, and the exploration of transplant effectiveness still needs further efforts (for example, shortening transplant time or times). Third, the incidence rate of ASD in males is four times that of females (53), which has a strong gender bias. However, our experiment only selects male offspring and lacks exploration of modeling gender differences. Fourth, we did not transplant normal human flora to eliminate the interference in the transplantation process, and the influence of normal human flora on offspring needs more experimental design to verify. The research on the relationship between gut microbiota and nervous system diseases is not only associated with ASD (2, 3, 7, 33, 50, 56, 57) but also in other neurological diseases such as depression and anxiety (58–60), epilepsy (61). Further studies are required on these aspects in the future. Our research may push the animal model of ASD to develop in another direction, which is of the great importance of the study of ASD.

## Conclusions

FMT autism rat model (oFMT) shows changes in neurotransmitter metabolism, and The FMT model is likely to be a new and reliable potential animal model of ASD.

## Data Availability Statement

The datasets presented in this study can be found in online repositories. The names of the repository/repositories and accession number(s) can be found at: https://www.ncbi.nlm.nih.gov/, SRP254427.

## Ethics Statement

The studies involving human participants were reviewed and approved by Institutional Review Board at The First Affiliated Hospital of Henan University of CM (2019 HL-084-02). Written informed consent to participate in this study was provided by the participants' legal guardian/next of kin. The animal study was reviewed and approved by Animal Ethics Committee of Henan University of Chinese Medicine (DWLL 2018030017).

## Author Contributions

ZQ performed the experiments and wrote the manuscript. ML and WD conducted the design of gut microbiota experiments and data analysis. LY provided financial support, guided the design of experiments, and revised the manuscript. HY, YC, LZ, JL, FY, and YL carried out experiments. YC, LZ, and YL guided experiments. HY and FY carried out data analysis. All authors contributed to the article and approved the submitted version.

## Conflict of Interest

The authors declare that the research was conducted in the absence of any commercial or financial relationships that could be construed as a potential conflict of interest.

## Abbreviations

ASD: Autism spectrum disorders
VPA: Valproic acid
5-HT: 5-hydroxytryptamine
FMT: Fecal microbiota transplantation
rFMT: FMT group maternal rats
oFMT: FMT group offspring rats
rVPA: VPA group maternal rats
oVPA: VPA group offspring rats
rCON: Control group maternal rats
rCON: Control group offspring rats
CDI: Clostridium difficile infection
CD: Crohn's disease
CM: Chinese Medicine
GABA: γ-aminobutyric acid
NE: norepinephrine
OTUs: Operational taxonomic units.
